# Accelerated Hypofractionated Active Raster-Scanned Carbon Ion Radiotherapy (CIRT) for Laryngeal Malignancies: Feasibility and Safety

**DOI:** 10.3390/cancers10100388

**Published:** 2018-10-18

**Authors:** Sati Akbaba, Kristin Lang, Thomas Held, Olcay Cem Bulut, Matthias Mattke, Matthias Uhl, Alexandra Jensen, Peter Plinkert, Stefan Rieken, Klaus Herfarth, Juergen Debus, Sebastian Adeberg

**Affiliations:** 1 Department of Radiation Oncology, University Hospital Heidelberg, Im Neuenheimer Feld 400, 69120 Heidelberg, Germany; sati.akbaba@med.uni-heidelberg.de (S.A.); kristin.lang@med.uni-heidelberg.de (K.L.); thomas.held@med.uni-heidelberg.de (T.H.); matthias.mattke@med.uni-heidelberg.de (M.M.); matthias.uhl@med.uni-heidelberg.de (M.U.); stefan.rieken@med.uni-heidelberg.de (S.R.); klaus.herfarth@med.uni-heidelberg.de (K.H.); juergen.debus@med.uni-heidelberg.de (J.D.); 2 Heidelberg Institute of Radiation Oncology (HIRO), Im Neuenheimer Feld 400, 69120 Heidelberg, Germany; 3 Heidelberg Ion-Beam Therapy Center (HIT), Im Neuenheimer Feld 450, 69120 Heidelberg, Germany; 4 Department of Otorhinolaryngology, Head and Neck Surgery, University Hospital Heidelberg, Im Neuenheimer Feld 400, 69120 Heidelberg, Germany; ocbulut@hotmail.com (O.C.B.); peter.plinkert@med.uni-heidelberg.de (P.P.); 5 Department of Radiation Oncology, University Hospital Giessen, Klinikstrasse 33, 35392 Giessen, Germany; alexdjensen@gmx.de

**Keywords:** carbon ion radiotherapy, adenoid cystic carcinoma, chondrosarcoma, laryngeal malignancies, laryngeal tumor, toxicity, bimodal RT

## Abstract

(1) Background: The authors present the first results of active raster-scanned carbon ion radiotherapy (CIRT) for radioresistant laryngeal malignancies regarding efficacy and toxicity. (2) Methods: 15 patients with laryngeal adenoid cystic carcinoma (ACC; *n* = 8; 53.3%) or chondrosarcoma (CS; *n* = 7; 46.7%) who underwent radiotherapy with carbon ions (C12) at the Heidelberg Ion Beam Therapy Center (HIT) between 2013 and 2018 were identified retrospectively and analyzed for local control (LC), overall survival (OS), and distant progression-free survival using the Kaplan–Meier method. CIRT was applied either alone (*n* = 7, 46.7%) or in combination with intensity modulated radiotherapy (IMRT) (*n* = 8, 53.3%). The toxicity was assessed according to the Common Toxicity Terminology Criteria for Adverse Events (CTCAE) v4.03. (3). Results: the median follow-up was 24 months (range 5–61 months). Overall, the therapy was tolerated very well. No grade >3 acute and chronic toxicity could be identified. The most reported acute grade 3 side effects were acute dysphagia (*n* = 2; 13%) and acute odynophagia (*n* = 3; 20%), making supportive nutrition via gastric tube (*n* = 2; 13.3%) and via high caloric drinks (*n* = 1; 6.7%) necessary due to swallowing problems (*n* = 4; 27%). Overall, chronic grade 3 toxicity in the form of chronic hoarseness occurred in 7% of the patients (*n* = 1; 7%). At the last follow-up, all the patients were alive. No local or locoregional recurrence could be identified. Only one patient with laryngeal ACC developed lung metastases three years after the first diagnosis. (4) Conclusions: the accelerated hypofractionated active raster-scanned carbon ion radiotherapy for radioresistant laryngeal malignancies is feasible in practice with excellent local control rates and moderate acute and late toxicity. Further follow-ups are necessary to evaluate the long-term clinical outcome.

## 1. Introduction

In recent years, more aggressive therapy regimes in the treatment of head and neck cancers (HNC) were established to achieve improved tumor control and prolonged survival, with the consequence of high toxicity rates due to the close proximity of organs at risk and increased doses applied [1,2,3]. Thus, a great number of HNC patients suffer from impaired functional outcome and quality of life after therapy [4,5,6,7,8,9]. Contrary to other tumors of the head and neck region, for laryngeal tumors, dose escalations, even with modern radiotherapy techniques—e.g., adapted intensity-modulated radiotherapy (IMRT)—seem to be limited by various critical structures and, consequently, the results in severe acute and chronic toxicity concerning airway obstruction, swallowing difficulties, and hoarseness [10,11,12]. To achieve an accurate dose escalation and, at the same time, a sufficient preservation of the organs at risk to minimize the treatment-related side effects, the use of carbon ions (C12) in the treatment of HNC represents a valuable alternative. Especially for mucosal melanoma, chordoma, chondrosarcoma, and adenoid cystic carcinoma of the head and neck, as well as for tumors with the skull base infiltration, e.g., advanced nasopharyngeal or sinonasal malignancies. Carbon ion radiotherapy (CIRT) shows promising results with improved local control rates and less toxicity compared to other modern radiotherapy techniques [9,13,14,15,16,17,18,19,20]. Prospective studies and long-term results for CIRT already exist for multiple entities [15,21]. However, the data for laryngeal carcinoma are missing to date. The aim of this analysis is to demonstrate the first results of CIRT alone or in combination with intensity modulated radiotherapy (IMRT) in a primary or postoperative setting as a further therapy option in the cancer treatment of the head and neck region, regarding the efficacy and toxicity for laryngeal malignancies. 

## 2. Results

### 2.1. Survival Analysis

The median follow-up was 24 months (range: 5–61 months). At the last follow-up, all patients were still alive (*n* = 15, 100%). No local or locoregional recurrence could be identified. The total laryngectomy was applied in one patient before radiotherapy (6.7%). Thus, in all other patients, we could provide organ-sparing (*n* = 14/15, 93%). Complete remission (CR) could be detected in three patients (20%), partial remission (PR) in five patients (33.3%), and stable disease (SD) in seven patients (46.7%). Before radiotherapy, the tracheostomy was obligatory for severe airway obstruction in 33.3% of the cases (*n* = 5). At the follow-up, these patients were no longer dependent on a tracheostoma due to a good tumor response. Lung metastases occurred three years after the first diagnosis (DC at last follow-up of 93%) in only one patient (with laryngeal adenoid cystic carcinoma (ACC)), who received bimodal radiotherapy (RT.)

### 2.2. Acute Toxicity

**Figure 1:**
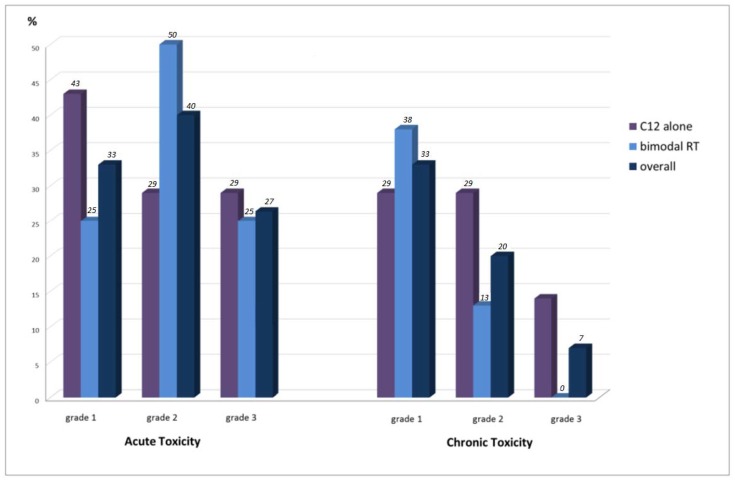
The distribution of CTCAE (Common Toxicity Criteria for Adverse Events) acute and chronic toxicity regarding C12 (carbon ions) alone vs. bimodal RT (radiotherapy) vs. overall. No grade >3 acute and late adverse side effects occurred. Overall, the chronic grade 3 toxicity was low (7%).

Treatment was well tolerated by the patients without any severe treatment-related side effects (Table 1, Figure 1). No grade 4 and 5 acute toxicity occurred. Grade 3 acute toxicity was reported in only four patients (26.7%). Acute grade 3 dysphagia could be identified in two cases (13.3%) and odynophagia in three cases (20%). Overall, 33.3% of the patients suffered from acute grade 1 (*n* = 5) and 40% from acute grade 2 toxicity (*n* = 6). The most identified acute grade 1/2 toxicities were dysphagia (*n* = 9; 60%), odynophagia (*n* = 9; 60%), dermatitis (*n* = 9; 60%), hoarseness (*n* = 8; 53.3%), xerostomia (*n* = 8; 53.3%), and mucositis (*n* = 6; 40%). Acute grade 1 alopecia was detected in five cases (33.3%) and dry cough in two cases (13.3%). Acute toxicity was equally distributed between CIRT alone and bimodal RT with IMRT and C12 boost. Supportive nutrition via a gastric tube was obligatory in two patients (13.3%) and via high caloric drinks in one patient (6.7%).

### 2.3. Chronic Toxicity

Chronic toxicity was rare. No chronic grade 4 and 5 toxicity could be identified. A chronic grade 3 toxicity in the form of hoarseness occurred 12 months post RT in only one patient (6.7%) with chondrosarcoma who received CIRT alone with doses up to 60 Gy (relative biological effectiveness (RBE))/3 Gy (RBE). Chronic grade 1 toxicity could be observed in 33% (*n* = 5) and grade 2 toxicity in 20% (*n* = 3) of the patients. Chronic grade 1 toxicity in the form of xerostomia (*n* = 8; 53.3%) and hoarseness (*n* = 5; 33.3%) dominated. Chronic grade 2 hoarseness was reported by three patients (20%), xerostomia by three patients (20%), and dysphagia by one patient (6.7%). According to the chronic toxicity, no relevant differences between CIRT alone and bimodal RT could be assessed. At the last follow-up, acute odynophagia, dermatitis, alopecia, and dry cough resolved in all the patients. No patient was dependent on supportive nutrition 12 months after therapy.

## 3. Discussion

The median follow-up was 24 months. At the last follow-up, no cases of local relapse or death appeared. Thus, the CIRT for laryngeal malignancies seemed to be feasible with excellent local control (LC) rates and OS despite limitations in the patient number, short follow-ups, and the retrospective character of the current study. Lung metastases occurred three years after therapy in only one patient with ACC. Larynx-preservation could be achieved in all patients who had not received prior laryngectomy before RT.

Treatment was tolerated well without any severe grade 4 or 5 acute and late side effects. Although a longer follow-up was needed to make a clear statement, late treatment-related side effects remained low. Thus, only one patient (7%) reported a chronic grade 3 toxicity in the form of hoarseness. We could not identify the major differences between CIRT alone and bimodal RT in the small study cohort. Both methods seem equally safe regarding toxicity. 

In a systematic research of the literature including the PubMed, Embase, and Cochrane Library databases, we could identify only a few retrospective analyses reporting the HNC patients who were treated with carbon ions [22,23,24,25,26,27]. Furthermore, we could not identify any cases of CIRT applied in laryngeal malignancies. Thus, the current study will be the first series reporting on CIRT either in combination with IMRT or alone for laryngeal malignancies. 

Dose-escalated radiotherapy with active raster-scanned carbon ions and intensity-modulated radiotherapy is an advanced method in the treatment of radioresistant tumors [14,15,28]. Long-term results regarding the use of bimodal RT with C12 for head and neck cancer show promising results in the LC and toxicity compared with photon radiotherapy [14,21,29]. Even for patients with head and neck tumors who received a local re-irradiation with C12, moderate toxicity was observed [22]. According to clinical outcomes, the recent data from the Heidelberg Ion Beam Therapy Center (HIT) showed a significantly higher local control and OS rate for radioresistant tumors treated with bimodal RT [15,21,29]. Jensen et al. described superior LC, progression-free survival (PFS), and OS for bimodal RT compared to IMRT for locally advanced ACC of the head and neck with a 5-year LC, PFS, and OS of 59.6%, 48.4%, and 76.5% vs. 39.9%, 27%, and 58.7% [14]. Uhl et al. reported about a 5-year LC and OS of 88% and 96.1% for chondrosarcome (CS) of the skull base treated with bimodal RT [28]. In recent years, these results could even be strengthened by prospective studies [15,16]. It is known that for head and neck cancer, the plan quality often suffers from inter-fractional anatomical changes possibly affecting the clinical outcome. Therefore, a cone beam CT for position correction was conducted routinely under RT. A re-planning was necessary due to position uncertainty in none of the cases. 

Higher prescribed doses to optimize tumor control for laryngeal malignancies are associated with increased swallowing dysfunction, stricture, and feeding tube dependence. These functional disorders correlate with the radiotherapy dose and the volume of surrounding irradiated tissue. Thus, an increase of the irradiated pharyngeal constrictor muscle volume >30% which receive >70 Gy lead to higher swallowing dysfunction and an irradiated pharyngeal constrictor muscle volume >50%, which receive more than 65 Gy to a higher aspiration rate [30,31,32]. Dysphagia especially occurs when the radiation exposure to the superior and middle pharyngeal constrictors exceeds 55 Gy and increases with every 10 Gy [33]. Carbon ions as heavy particles are known for achieving excellent dose conformity in deep-seated tumors with higher relative biological effectiveness compared with protons or photons and a maximum preservation of the surrounding tissue due to its biological and physical advantages. Therefore, C12 could be a notable strategy for lessening the treatment morbidity in the radiotherapy of laryngeal malignancies while reducing the dose and volume of normal tissue irradiated. 

It is difficult to compare our results with other carbon ion experiences for head and neck cancers because of the missing data for laryngeal malignancies, different tumor sites, and various dose calculation models for CIRT reported in the current literature. Nevertheless, several authors describe similar toxicity rates for CIRT in the head and neck regions with insignificant high-grade acute and chronic side effects [13,14,15,16,18,19,20,21,28,29,34]. The long-term results for bimodal RT for the ACC of the head and neck by Jensen et al. and for the CS of the skull base by Uhl et al. strengthen our findings with reports of similar side effects for this therapy regime [21,28]. Compared with photon radiotherapy, CIRT tends to decrease the toxicity [13]. Prospective studies comparing both therapy alternatives for head and neck cancers are still missing. Data regarding IMRT for the head and neck cancer showed a superior dose conformity and improved preservation of organs at risk with decreased toxicity rates compared with 3D-radiotherapy [35,36,37,38,39,40]. Nevertheless, in the hypopharynx and larynx region, IMRT can lead to severe acute and chronic side effects due to the proximity of several organs at risk [11,12,41,42,43]. In a meta-analysis of three prospective randomized Radiation Therapy Oncology Group (RTOG) studies, Machtay et al. identified the larynx and hypopharynx as independent negative prognostic factors for severe late side effects after concurrent chemoradiotherapy with IMRT [12]. The most described late side effects were laryngoesophageal dysfunction with swallowing problems and aspiration, laryngoesophageal stricture, and dysphagia. Thus, Ward et al. reported on a 5-year occurrence of severe dysphagia in 27% of the patients who were treated by chemoradiotherapy with IMRT for locally advanced stage laryngeal tumors [41]. Caudell et al. and Chen et al. described a high rate of severe laryngoesophageal dysfunction after chemoradiotherapy for locally advanced stage hypopharyngeal and laryngeal tumors [42,43]. It is known that concurrent chemotherapy in combination with radiotherapy led to an increased toxicity compared with radiotherapy alone [11]. Thus, a direct comparison of these studies with the current study (CIRT without concurrent systemic therapy) is difficult. However, Forastiere et al. showed in an RTOG trial 91–11 the severe toxicity rates for the larynx and the pharynx/esophagus even after larynx-preserving IMRT without concurrent chemotherapy, with 49% grade 3, 10% grade 4, and 0.6% grade 5 toxicity for patients who received radiotherapy alone [11]. In comparison, in the current analysis, no laryngoesophageal dysfunction or stricture could be identified for CIRT. Although altered fractionation regimes like hypofractionated IMRT results in significantly higher toxicity rates, accelerated hypofractionated CIRT seems to be a safe therapy alternative without any severe late effects [43,44,45]. For further evaluation, long-term follow-ups are necessary.

## 4. Materials and Methods 

Medical records of fifteen patients who received CIRT either alone or in combination at the Heidelberg Ion Beam Therapy Center (HIT) and the Department of Radiation and Oncology of the University Hospital Heidelberg were identified and analyzed retrospectively. The study was conducted in accordance with the Declaration of Helsinki, and the protocol was approved by the Ethics Committee of the University Hospital Heidelberg (S-421/2015).

Before radiotherapy, a clinical examination, the Eastern Cooperative Oncology Group (ECOG) performance status, as well as preserving symptoms, were assessed in all patients by an otorhinolaryngologist. Endoscopy, computed tomography (CT), and magnetic resonance imaging (MRI) were performed to determine the tumor, nodal, and metastasis stage according to the 8th edition of the Union for International Cancer Control (UICC) TNM classification guidelines.

### 4.1. Treatment Planning 

**Figure 2:**
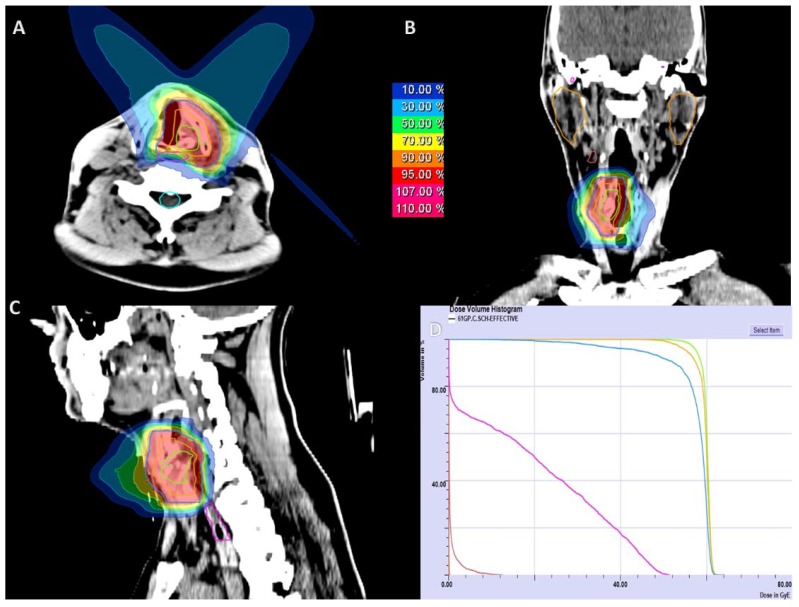
The active raster-scanned carbon ion radiotherapy (CIRT) alone in a patient with chondrosarcoma of the left vocal cord. CIRT was applied with two beams at 60 Gy (relative biological effectiveness, RBE) in 3 Gy (RBE) per fraction. (**A**) Axial dose distribution; (**B**) coronal dose distribution; (**C**) sagittal dose distribution; (**D**) dose-volume histogram: gross tumor volume (GTV) is demonstrated in green color, clinical target volume (CTV) in orange, CTV including a 5 mm safety margin in blue, the tracheoesophageal junction in pink, and the submandibular salivary gland in brown color.

Treatment planning was performed based on a non-contrast CT scan with a slice thickness of 3 mm in the irradiation position. For immobilization, an individual thermoplastic head mask with a shoulder fixation was used. Target delineation based on a contrast-enhanced CT or MRI was performed for image correlation (SyngoVia, VB20, 2017, Siemens, Erlangen, Germany). The gross tumor volume (GTV) was defined as the delineated primary tumor. CTV1 (clinical target volume 1) involved the macroscopic tumor with a safety margin of 5 mm including areas of possible microscopic spread for definite radiotherapy or the whole larynx for postoperative radiotherapy. In case of ACC histology, CTV2 for the lymphatic drainage was delineated. The planning target volume (PTV) included the CTV with a safety margin of 3–5 mm. Chondrosarcomas were irradiated with carbon ions only (*n* = 7; 47%) up to 60 Gy (RBE, relative biological effectiveness) in 3 Gy (RBE) single dose fractions. Tumors of ACC histology (*n* = 8; 53%) received bimodal RT with IMRT doses of 50 Gy to 54 Gy in 2 Gy single dose fractions to the CTV2 and a carbon ion boost to the CTV1 with 18 Gy (RBE) to 24 Gy (RBE) in 3 Gy (RBE) single dose fractions. CTV2 received at least 90% and CTV1 received at least 95% of the prescription isodose. CIRT was applied in 5–6 fractions per week at the Heidelberg Ion Beam Therapy Center (HIT) or at the Marburg Ion Beam Therapy Center (MIT) with active raster-scanning and daily position correction. IMRT was applied in 5 fractions per week with a daily portal image guidance and weekly performed MV-CT scans for position correction. Dose constraints to the constrictor muscles and to the esophageal constrictor were defined as low as reasonably available. Doses for the spinal cord were limited to 45 Gy (EQD2). A treatment plan showing active raster-scanned radiotherapy alone for a patient with chondrosarcoma of the left vocal cord is depicted in Figure 2. For improved comparability, the equivalent dose in 2 Gy per fraction was calculated using the formula EQD2 = D × ((d + α/β)/(2 + α/β)) (D = total dose in Gy; d = fraction dose in Gy; α/β = 2). 

### 4.2. Follow-up

Follow-up was initiated 6 weeks after the completion of radiotherapy, every three months during the first two years, every six months during the third year and, subsequently, once a year after the MRI treatment. A clinical examination was performed every six weeks within the first two years after treatment and then every 3 months. Once a year, a CT scan of the lungs was performed to exclude distant metastases. The tumor response was captured according to the Response Evaluation Criteria in Solid Tumors (RECIST) [46]. Complete response (CR) was defined as a disappearance of the tumor in response to the treatment, partial response (PR) as a decrease of the tumor size by >30% in response to the treatment, and stable disease (SD) when the tumor size neither decreased nor increased by >20% after the treatment. Acute and late toxicity was assessed according to the Common Toxicity Terminology Criteria for Adverse Events (CTCAE) v4.03. 

### 4.3. Analysis

Statistical analyses were performed with IBM SPSS Statistics version 24 (International Business Machines Corporation, Armonk, NY, USA) using the Kaplan–Meier method for survival analysis. OS, LC rate, and treatment-related acute (up to 3 months after treatment), as well as chronic toxicity (>3 months after treatment), were analyzed. Local control was assessed from the day of commencement of RT to the day of local progressive disease (PD). OS was considered as the time period between the first diagnosis and last follow-up or death. DC was assessed from the day of therapy commencement to distant tumor progression.

### 4.4. Patient, Disease, and Treatment Characteristics

Fifteen patients with a laryngeal malignancy treated with CIRT at our department were identified using the National Center for Tumor Disease (NCT) cancer registry. Patient, disease, and treatment characteristics are shown in Table 2. The primary tumor types were ACC in 53.3% (*n* = 8) and CS in 46.7% (*n* = 7) of the patients. The most common tumor sites were the supraglottic (*n* = 7; 46.7%) and the glottic larynx (*n* = 7; 46.7%). In only one case was the tumor located in the subglottic larynx (6.7%). Initial symptoms at the time of the first diagnosis were hoarseness (*n* = 7; 46.7%), dyspnea (*n* = 5; 33.3%) and dysphagia (*n* = 3; 20%). The majority of the tumors were locally advanced with the T3 stage having been reached by two patients (13.3%), the T4a stage by six patients (40%), and the T4b stage by two patients (13.3%). At the time of the first diagnosis, no patient showed lymph node involvement (N0) or distant metastases (M0). According to the grading, three patients could not be staged for grading (20%), five patients were staged G1 (33.3%), three patients were G2 (20%), and four patients were G3 (26.7%). According to the patient and disease characteristics, no significant differences between CIRT alone and bimodal RT could be detected.

Radiotherapy was applied postoperatively in six cases (40%) and definitely in nine cases (60%). Overall, one patient received laryngectomy (overall 6.7%) and five patients received a larynx-sparing laser surgical partial tumor resection (overall 33.3%) before radiotherapy. Negative resection margins could be achieved in only one patient with a total laryngectomy (6.7%), R1 in another patient (6.7%) and R2 in four patients (26.7%). Functional neck dissection was applied in three patients (20%). Only one patient with laryngeal ACC received concomitant cetuximab 75 mg/m² weekly (6.7%); 7 cycles. The majority of the patients who received prior surgery were treated with CIRT alone (57%; *n* = 4). Overall, 53.3% of the patients received CIRT in combination with IMRT (*n* = 8) and 46.7% received CIRT alone (*n* = 7). The prescribed doses were 50 Gy in a 2 Gy single dose fraction for IMRT and 18 Gy (RBE) (*n* = 4; 26.7%) or 24 Gy (RBE) (*n* = 4; 26.7%) in a 3 Gy (RBE) single dose fraction for the C12 boost and 60 Gy (RBE) in a 3 Gy (RBE) single dose fraction for CIRT alone. The median EQD2 (equivalent dose to 2 Gy ) was 75 Gy for all patients (range: 72.5–80 Gy). The median PTV volume prescribed on the primary tumor was 110 cc (range: 59–150 cc) for C12 alone and 500 cc (range: 387–731 cc) for the bimodal RT with a median boost volume of 118 cc (62–138 cc) for the C12 boost. The median follow-up was 24 months (range: 8–61 months).

## 5. Conclusions

Accelerated hypofractionated active raster-scanned carbon ions radiotherapy for laryngeal malignancies seemed to be feasible and safe in practice with excellent local control rates and moderate side effects. Especially for radioresistant tumors of the head and neck, carbon ions seem to be an effective method for dose escalation. Further follow-ups are necessary to evaluate the long-term clinical outcome.

## Figures and Tables

**Table 1:** An overview of the acute and chronic toxicities.

Characteristic	Acute Toxicity, No. (%)			Chronic Toxicity, No. (%)		
(*n* = 15)	C12 Alone (*n* = 7)	IMRT + C12 (*n* = 8)	Total (*n* = 15)	C12 Alone (*n* = 7)	IMRT + C12 (*n* = 8)	Total (*n* = 15)
overall toxicity						
grade 1	3 (43)	2 (25)	5 (33)	2 (29)	3 (38)	5 (33)
grade 2	2 (29)	4 (50)	6 (40)	2 (29)	1 (13)	3 (20)
grade 3	2 (29)	2 (25)	4 (27)	1 (14)	0	1 (7)
grade 4	0	0	0	0	0	0
dysphagia						
grade 1	3 (43)	3 (38)	6 (40)	3 (43)	0	*n* = 3 (20)
grade 2	1 (14)	2 (25)	3 (20)	1 (14)	0	*n* = 1 (7)
grade 3	1 (14)	1 (13)	2 (13)	0	0	0
odynophagia						
grade 1	4 (57)	3 (38)	7 (53)	0	0	0
grade 2	1 (14)	1 (13)	2 (13)	0	0	0
grade 3	2 (29)	1 (13)	3 (20)	0	0	0
mucositis						
grade 1	2 (29)	0	2 (13)	0	0	0
grade 2	1 (14)	3 (38)	2 (13)	0	0	0
dermatitis						
grade 1	3 (43)	3 38 ()	6 (40)	0	0	0
grade 2	0	3 (38)	3 (20)	0	0	0
xerostomia						
grade 1	3 (43)	4 (50)	7 (53)	5 (71)	3 (38)	8 (53)
grade 2	1 (14)	0	1 (7)	0	3 (38)	3 (20)
hoarseness						
grade 1	2 (29)	3 (38)	5 (33)	2 (29)	3 (38)	5 (33)
grade 2	2 (29)	1 (13)	3 (20)	2 (29)	1 (13)	3 (20)
grade 3	0	0	0	1 (14)	0	1 (7)
fatigue						
grade 1	0	2 (25)	2 (13)	2 (29)	1 (13)	3 (20)
grade 2	4 (57)	0	4 (27)	1 (14)	0	1 (7)
grade 3	0	1 (13)	1 (7)	0	0	0
dysgeusia						
grade 1	0	2 (25)	2 (13)	1 (14)	1 (13)	2 (13)
grade 2	0	2 (25)	2 (13)	0	0	0
alopecia						
grade 1	3 (43)	2 (25)	5 (33)	0	0	0
dry cough						
grade 1	1 (14)	1 (13)	2 (13)	0	0	0

Abbreviations: C12 = carbon ions, IMRT = intensity-modulated RT.

**Table 2:** The patient, disease, and treatment characteristics.

Characteristic	C12 Alone, *n* = 7 No. (%)	C12 + IMRT, *n* = 8 No. (%)	Total, *n* = 15 No. (%)
median age	61 years (55–73 years)	58 years (21–68 years)	61 years (21–73 years)
median follow-up	22 months (9–48 months)	24 months (8–61 months)	24 months (8–61 months)
gender			
male	3 (43)	4 (50)	7 (47)
female	4 (57)	4 (50)	8 (53)
WHO performance status			
0	4 (57)	4 50 ()	8 (53)
1	3 (43)	3 (38)	6 (40)
2	0	1 (12)	1 (7)
tumor type			
ACC	0	8 (100)	8 (53)
CS	7 (100)	0	7 (47)
tumor site			
glottic larynx	2 (29)	4 (50)	7 (47)
subglottic larynx	1 (14)	0	1 (7)
supraglottic larynx	4 (57)	4 (50)	7 (47)
initial T stage			
T1	2 (29)	1 (12)	3 (20)
T2	1 (14)	1 (12)	2 (13)
T3	2 (29)	0	2 (13)
T4a	1 (14)	5 (63)	6 (40)
T4b	1 (14)	1 (12)	2 (13)
initial N stage			
N0	7 (100)	8 (100)	15 (100)
N+	0	0	0
initial M stage			
M1	7 (100)	8 (100)	15 (100)
M0	0	0	0
treatment			
RT only	3 (43)	6 (75)	9 (60)
postop. RT	4 (57)	2 (25)	6 (40)
total laryngectomy	0	1 (12)	1 (7)
laser surgical resection	4 (57)	1 (12)	5 (33)
concomitant cetuximab	0	1 (12)	1 (7)
median EQD2 in Gy	75 Gy (75–75 Gy)	76.25 Gy (72.5–80 Gy)	75 Gy (72.5–80 Gy)

Abbreviations: C12 = carbon ions, IMRT = intensity-modulated RT, WHO = World Health Organization, ACC = adenoid cystic carcinoma, CS = chondrosarcoma, TNM stage = tumor, nodal, metastasis stage, RT = radiotherapy, postop. = postoperative, EQD2 = equivalent dose to 2 Gy.
